# Factors influencing quality of professional life and perceived bullying among medical residents in Iran

**DOI:** 10.1186/s12909-025-06724-8

**Published:** 2025-02-11

**Authors:** Nima Assadpour, Farshid Alazmani-noodeh, Faeze Baniyaghoobi, Zahra Shafiei Kisomi, Mahdie Bahrami, Seideh Madineh Ghasemnegad, Hadi Ranjbar

**Affiliations:** 1 Iran Univerity of Medical Sciences, Tehran, Iran; 2 School of Nursing, AJA University of Medical Sciences, Tehran, Iran; 3 Department of Nursing, Lahijan Branch, Islamic Azad University, Lahijan, Iran; 4 Mental Health Research Center, Psychosocial Health Research Institute, Iran University of Medical Sciences, Tehran, Iran; 5 PhD Student in Nursing, Tehran University of Medical Sciences, Tehran, Iran; 6 PhD Student in Nursing, Qazvin University of Medical Sciences, Qazvin, Iran

**Keywords:** Healthcare System, Workplace bullying, Medical residents, Quality of life

## Abstract

**Objectives:**

During residency training, medical doctors, face significant challenges that can impact their quality of professional life, their performance, and the care they provide. The current study aimed to examine factors affecting the quality of professional life and their perception of bullying.

**Design/ participants:**

This was a cross-sectional study with 231 medical residents.

**Setting:**

Educational hospitals affiliated with Iran University of Medical Sciences in Tehran, Iran.

**Methods:**

Participants completed a questionnaire that included demographic data, the Negative Act Questionnaire-Revised (NAQ-R), and the Professional Quality of Life Scale (ProQol-5). The study included 231 medical residents from medical university-affiliated hospitals.

**Results:**

Bullying had a significant negative effect on the quality of professional life (B = -1.173, *p* < 0.001), while satisfaction with income positively affected it (B = 2.111, *p* = 0.016). Gender, marital status, feeling respected, age, shift hours, residency year, and medical-surgical department were not significant predictors. Longer shift hours per week (B = 0.306, *p* = 0.044) and more experienced residents (B = -1.376, *p* = 0.010) were associated with higher and lower bullying perceptions, respectively.

**Conclusions:**

The study highlights the significant negative impact of bullying on the quality of professional life among medical residents. Suitable interventions should be considered to reduce this impact. Potential interventions could include implementing comprehensive training programs focused on emotional intelligence and communication skills, establishing clear reporting mechanisms for bullying incidents, and fostering a supportive workplace culture prioritizing respect and collaboration among healthcare professionals.

## Key message

Workplace bullying negatively affects the quality of professional life among medical residents, negatively impacting their performance and the care they provide.

Satisfaction with income positively affects the quality of professional life among medical residents, indicating that financial stability can contribute to a better professional life.

Working long shift hours per week is associated with a higher bullying perception suggesting that long hours of work can negatively affect doctors’ dignity.

## Introduction

Workplace bullying is defined as the repetitive and systematic engagement of interpersonally abusive behaviors that negatively affect both the targeted individual and the organization [1, 2]. It is a persistent form of impairment that arises in all industries, including healthcare, and is defined as repeated, unreasonable behavior directed at an employee or a group of employees that poses a risk to their health and safety [3]. Types of Workplace bullying include verbal abuse, intimidation, exclusion, and sabotage. In the medical profession, workplace bullying is common and is of particular concern given its implications for the well-being of doctors and the quality of patient care [4, 5].

The medical training environment can be particularly stressful [6], especially during medical education residency [7]. There is a significant amount of evidence about the high rate of bullying in medical education worldwide [8]. Studies in Iran like other countries showed that medical residents are under much pressure including intimidation, harassment, and discrimination (IHD) [9, 10]. In Iranian medical education culture, the hierarchical power structures and stressful workloads inherent to medical training, particularly during residency, may provide the ideal environment for the occurrence of workplace bullying [11, 12]. Studies in Iran also showed that the consequences of workplace bullying on medical residents include increased stress, burnout, and mental health issues [13, 14]. Residents who have been bullied may also be less effective in their clinical duties, compounding the potential for harm to patients [15–17].

Workplace bullying among medical residents is a significant issue that has been explored in various studies there is a notable gap in research specific to the Iranian context. While there is evidence of the high prevalence of bullying its effect on professional quality of life was not assessed.

F Ajoudani, R Baghaei and M Lotfi [18] In Iran, specific cultural factors—such as deeply entrenched hierarchical structures in medical education, low income, and unclear work future—may exacerbate the prevalence of workplace bullying among medical residents in Iran. The traditional respect for authority within Iranian culture can discourage open communication about bullying experiences and hinder reporting mechanisms. Additionally, systemic issues such as high patient loads in teaching hospitals and insufficient institutional support further contribute to an environment where bullying can thrive.

Workplace bullying among medical residents is a global problem [19]. Features of teaching hospitals characterized by hierarchical systems may provide a conducive environment for the occurrence of bullying [20]. In Iran, the high-stress nature of clinical training and the lack of institutional support along with finantial and emotional problem make trainees an easy target for bullying [20]. Identifying and understanding the risk factors for bullying specific to residents is an important step in identifying goals for interventions in medical education. We believe that understanding the important factors associated with bullying and its negative impact on the quality of work in public hospitals and then resolving these issues will be beneficial in establishing a culture of respect for human dignity and work in teaching hospitals. The current study was conducted to determine the prevalence and specific factors related to workplace bullying among medical doctors in residency training in Iran and explore the impact of bullying on medical residents’ professional quality of life.

## Methods

### Design

This cross-sectional study was conducted from April 2022 to March 2023.

### Subjects and settings

The study involved medical residents from diverse specialties within educational hospitals affiliated with Iran University of Medical Sciences. The inclusion criteria for the study were medical residents currently enrolled in residency programs at educational hospitals affiliated with Iran University of Medical Sciences, ensuring representation from various specialties to capture diverse perspectives. Participants were required to express their willingness to participate in the study. The exclusion criteria consisted of residents not actively engaged in their programs at the time of the research and those who declined to participate or did not complete the questionnaire. The study population consisted of 1153 medical residents. Based on the study’s aims, 300 (25%) medical residents were reached based on demographic information such as age, gender, years of residency, and their willingness to participate to ensure a comprehensive understanding of the sample characteristics. From 300 questionnaires distributed, 231 (77%) medical residents returned their answers and participated in the study.

### Data collection

An online electronic questionnaire was used as the primary tool for data collection which was comprised of three sections:

- Demographic Data: Gathering information on age, gender, and years of residency.
- Negative Act Questionnaire-Revised (NAQ-R).

The Negative Acts Questionnaire-Revised (NAQ-R) is a is a 22-item measure for evaluating workplace bullying by measuring the frequency of exposure to negative behaviors. The measure covers three underlying factors: personal bullying, work-related bullying, and physically intimidating forms of bullying. The NAQ-R has been translated into numerous languages and applied in various cultural contexts such as Malaysia, Estonia, India, and Spain. Multiple studies have extensively examined its psychometric properties. For instance, research among Malaysian junior doctors demonstrated the NAQ-R’s validity and reliability, revealing a one-factor structure labeled “workplace bullying” with high internal consistency (Cronbach’s alpha of 0.97) and robust test-retest reliability (ICC for NAQ-R total score at 93.4%). The scale’s total score reflects the overall exposure level to bullying behaviors. Higher total scores indicate a greater frequency of experienced negative acts, suggesting a more severe impact on the individual’s work environment and psychological well-being. The NAQ-R is designed to measure anxiety levels specifically in nursing and medical contexts, making it suitable for residents who often experience high-stress environments [21–23]. It has been validated in various healthcare settings, including studies conducted in Iran, which demonstrate its effectiveness in assessing anxiety among healthcare professionals [24, 25].

- The Professional Quality of Life Scale (ProQol-5).

The Professional Quality of Life Scale (ProQol-5) is a commonly utilized tool for assessing the quality of work life in healthcare professionals. It gauges three dimensions: compassion satisfaction, burnout, and secondary traumatic stress, encompassing 30 items with 10 items dedicated to each dimension. Employing a 5-point Likert scale, the ProQol-5 has been translated into various languages, including Persian, Greek, and Portuguese, and has seen application in diverse healthcare settings. Psychometric evaluations have been conducted globally, revealing satisfactory results. Higher scores on this scale represent a greater satisfaction related to the ability to be effective in the job.

The questionnaires were distributed to medical residents in a paper-based format to facilitate ease of use and ensure higher response rates. Printed questionnaires were delivered directly to the residency program offices and made available to residents during scheduled meetings and training sessions. We provided secure drop boxes in the residency offices for residents to submit their completed questionnaires anonymously, ensuring confidentiality and encouraging honest responses. The ProQOL-5 captures the dimensions of compassion satisfaction, burnout, and secondary traumatic stress, which are critical factors influencing the well-being of medical residents in healthcare system [26].

### Statistical analyses

Data was analyzed using SPSS 16. Descriptive statistics were calculated for all study variables, summarizing the data with means, standard deviations, and frequency distributions to provide an overview of the sample population. Data were analyzed using linear regression to examine the relationship between bullying experiences and Quality of Work Life, along with other predictors. This analysis quantified both direct and interaction effects among the variables of interest. The significance of the regression coefficients was evaluated using p-values, with a threshold set at *p* < 0.05 to determine statistical significance.

## Results

The demographic characteristics of study participants are presented in Table 1. Must study subjects were male, married, and learning in medical fields. The satisfaction with income and being respected was low. Their average age was above 30 years, and their weekly shift hours were above 54. Table 2 presents the predictors of professional quality of life among medical residents. Based on the linear regression bullying had a significant negative effect on the quality of professional life and higher levels of bullying were associated with lower quality of professional life. It means that a sense of being bullied caused a decrease in their quality of life. Satisfaction with income had a significant positive effect on the quality of professional life, which means higher income was related to better quality of professional life. The predictors of bullying perception are presented in Table 3. Based on the linear regression analysis results. Gender, marital status, age, and satisfaction with income were not predictors of the perception of bullying. Residents who feel more respected, with lower shift hours and higher residency years experienced lower levels of bullying.

**Table 1:** Demographic characteristics of study participants

		*N*	Percentage	Mean (Standard Deviation)	
Gender	Male	156	67.5%		
	Female	75	32.5%		
Marital	Single	105	45.5%		
	Married	126	54.5%		
Major	Medical	125	54.1		
	Surgical	106	45.9		
Satisfaction with income (1–5)				2.65 ± 1.15	
Feeling Respected (1–5)				3.01 ± 1.45	
Age				32.48 ± 3.68	
Shift hours per week				54.61 ± 3.99	
Residency Year				2.41 ± 1.11	

**Table 2:** Predictors of Professional Quality of Life among healthcare workers

Model	Unstandardized Coefficients		Standardized Coefficients		
	B	Std. Error	Beta	t	Sig.
(Constant)	176.881	17.415		10.157	0.000
Bullying	-1.173	0.080	-0.724	-14.715	0.000
Gender (Female to Male)	0.948	2.118	0.020	0.448	0.655
Marital (Married to Single)	-1.665	1.961	-0.038	-0.849	0.397
Satisfaction with income	2.111	0.871	0.111	2.426	0.016
Feeling Respected	-0.030	0.682	-0.002	-0.045	0.965
Age	0.065	0.262	0.011	0.247	0.805
Shift hours per week	-0.271	0.260	-0.049	-1.040	0.299
Residency Year	0.241	0.901	0.012	0.267	0.790
Medical-Surgical (Medical to Surgical)	-1.566	1.928	-0.036	-0.812	0.418

a. Dependent Variable: Quality of professional life

**Table 3:** Predictors of bullying among healthcare workers

Model		Unstandardized Coefficients		Standardized Coefficients		
		B	Std. Error	Beta	t	Sig.
1	(Constant)	89.080	11.175		7.971	0.000
	Gender	2.319	1.260	0.080	1.840	0.067
	Marital	-1.695	1.183	-0.062	-1.433	0.153
	Satisfaction with income	-0.229	0.529	-0.019	-0.432	0.666
	Feeling Respect	0.788	0.412	0.082	1.911	0.057
	Age	-0.033	0.157	-0.009	-0.209	0.834
	Shift hours per week	0.306	0.151	0.090	2.021	0.044
	Residency Year	-1.376	0.532	-0.113	-2.588	0.010
	Medical Surgical	-0.194	1.158	-0.007	-0.167	0.867
	Quality of life	-0.436	0.029	-0.706	-15.228	0.000

a. Dependent Variable: Bullying

## Discussion

This study aimed to examine the factors influencing the quality of professional life and the perception of bullying among medical residents in Iran. Our results showed that bullying negatively impacts the quality of professional life among medical residents in Iran, while satisfaction with income positively influences it.

The findings of this study align with previous studies on the impact of bullying on the quality of professional life of medical residents. The results of a study by J Peng, H Luo, Q Ma, Y Zhong, X Yang, Y Huang, X Sun, X Wang, J He and Y Song [27] on nurses showed that workplace bullying had a negative and direct effect on the professional quality of life of nurses, while resilience was a protective factor that helped nurses cope with workplace bullying. Another study by NV Fabri, JT Martins, MJQ Galdino, RP Ribeiro and AAO Moreira [28] showed that bullying was related to lower quality of life among nurses. The results of a study in Mexico showed that violence and bullying was very common among medical residents and it was related to a lower level of quality of life [29]. The results of a study on pediatric residents in the US showed that bullying was frequent and related to lower levels of quality of life [17]. These results suggest that bullying is a global problem in medical settings, especially in the field of medical education.

Our results showed that satisfaction with income was related to a low level of quality of professional life. Results of a study in Iran on healthcare personnel also showed that working conditions and income were related to lower levels of quality of professional life [30]. Income is an important predictor of professional quality of life because it can affect the resilience of healthcare providers [31, 32]. Medical residents are not among the well-paid professions in Iran which may hurt their sense of self-worth and also vulnerable them to bullying.

Our results also showed that weekly hours of work increased the sense of being bullied and lower residency year was also a predictor of being bullied. The hierarchical nature of medical education in Iran and globally is a predictor of bullying in the workplace [15, 33]. In the context of medical education in Iran, our results complied with previous studies. Medical residents in Iran suffer from long shifts and low income which can cause burnout and dissatisfaction and it was mentioned in previous studies [20]. While the hierarchical structure of medical education in Iran is comparable to other countries, we can not compare the economic status and hard work situation of medical residents in Iran with other countries. The traditional hierarchical structure of medical education in Iran puts a lot of pressure on medical residents and the nursing shortage also causes harder work and bad living status among medical residents, which accompanies long shift hours and low income.

### Strengths and limitations of the study

The main strength of the current study is that it provides valuable insights into the impact of bullying on the quality of professional life among medical residents, which is a critical issue in the healthcare sector. Also, the study’s inclusion of factors such as satisfaction with income and shift hours per week as predictors of quality of professional life and bullying perception adds depth to the understanding of these relationships. However, the study has some limitations. One of the primary limitations is the potential for bias in the self-reported data collected from the participants. Additionally, the study’s reliance on a cross-sectional design may not capture the longitudinal effects of bullying on the quality of life of medical residents. Furthermore, the study’s sample size was relatively small, which may not be representative of the broader population of medical residents in Iran. Finally, the study’s findings may not be generalizable to other countries or cultures, which could limit the applicability of the results. One of the primary limitations is the potential for bias in the self-reported data collected from the participants. Self-reported data can introduce various biases, including social desirability bias, where respondents may provide answers they believe are more acceptable rather than their true feelings or experiences. To enhance methodological rigor in future studies, alternative methods such as semi-structured interviews or focus groups could be employed.

## Conclusion

Our findings indicate that bullying has a detrimental impact on the quality of professional life for medical residents, while satisfaction with income serves as a positive influence. Our results underscore the need for medical institutions to address bullying proactively, as it not only affects individual well-being but also influences the overall work environment and patient care. To alleviate the negative effects of bullying, healthcare organizations should implement comprehensive training programs that promote awareness and foster respectful relationships among staff. Additionally, enhancing income satisfaction through improved compensation and benefits can lead to greater job satisfaction among residents. Reevaluating shift lengths to ensure a better work-life balance and establishing mentorship programs for junior residents can further support a positive work culture.

To strengthen these efforts, medical institutions must establish clear reporting mechanisms and implement supportive policies that explicitly prohibit bullying behaviors. Regular assessments should be conducted to monitor the workplace environment and identify any instances of bullying, ensuring that appropriate interventions are applied promptly. Fostering an organizational culture that encourages open communication can empower residents to voice concerns without fear of reprisal, thereby enhancing accountability among staff members.

## Data Availability

The data supporting the findings of this study are available upon reasonable request from the corresponding author.

## Abbreviations

NAQ-R: Negative Act Questionnaire-Revised
